# Dopamine transporter neuroimaging accurately assesses the maturation of dopamine neurons in a preclinical model of Parkinson’s disease

**DOI:** 10.1186/s13287-020-01868-4

**Published:** 2020-08-08

**Authors:** Julian L. Goggi, Lifeng Qiu, Mei Chih Liao, Shivashankar Khanapur, Lingfan Jiang, Ramasamy Boominathan, Siddesh V. Hartimath, Peter Cheng, Fui Fong Yong, Vanessa Soh, Xiaozhou Deng, Youshan Melissa Lin, Anna Haslop, Peng Wen Tan, Xiaoxia Zeng, Jolene W. L. Lee, Zhiwei Zhang, Pragalath Sadasivam, Eng King Tan, Sajinder K. Luthra, William D. Shingleton, Steve K. W. Oh, Li Zeng, Edward G. Robins

**Affiliations:** 1 Singapore Bioimaging Consortium, A*STAR, 11 Biopolis Way, #01-02 HELIOS, Singapore, 138667 Singapore; 2 Neural Stem Cell Research Lab, Research Department, National Neuroscience Institute, 11 Jalan Tan Tock Seng, Singapore, 308433 Singapore; 3 Bioprocessing Technology Institute, A*STAR, 20 Biopolis Way, #06-01 Centros, Singapore, 138668 Singapore; 4 Research Department, National Neuroscience Institute, SGH Campus, Singapore, 169856 Singapore; 5 Department of Neurology, National Neuroscience Institute, SGH Campus, Singapore, 169856 Singapore; 6 Neuroscience & Behavioural Disorders Program, DUKE-NUS Graduate Medical School, Singapore, 169857 Singapore; 7 GE Healthcare Life Sciences, White Lion Rd., Little Chalfont, Amersham, HP7 9LL UK; 8 Lee Kong Chian School of Medicine, Novena Campus, 11 Mandalay Road, Singapore, 308232 Singapore; 9 Clinical Imaging Research Centre, Yong Loo Lin School of Medicine, National University of Singapore, Singapore, 117599 Singapore

**Keywords:** Parkinson’s disease, Dopaminergic neuron, Cell therapy, PET imaging, DAT

## Abstract

**Background:**

Significant developments in stem cell therapy for Parkinson’s disease (PD) have already been achieved; however, methods for reliable assessment of dopamine neuron maturation in vivo are lacking. Establishing the efficacy of new cellular therapies using non-invasive methodologies will be critical for future regulatory approval and application. The current study examines the utility of neuroimaging to characterise the in vivo maturation, innervation and functional dopamine release of transplanted human embryonic stem cell-derived midbrain dopaminergic neurons (hESC-mDAs) in a preclinical model of PD.

**Methods:**

Female NIH RNu rats received a unilateral stereotaxic injection of 6-OHDA into the left medial forebrain bundle to create the PD lesion. hESC-mDA cell and sham transplantations were carried out 1 month post-lesion, with treated animals receiving approximately 4 × 10^5^ cells per transplantation. Behavioural analysis, [^18^F]FBCTT and [^18^F]fallypride microPET/CT, was conducted at 1, 3 and 6 months post-transplantation and compared with histological characterisation at 6 months.

**Results:**

PET imaging revealed transplant survival and maturation into functional dopaminergic neurons. [^18^F]FBCTT-PET/CT dopamine transporter (DAT) imaging demonstrated pre-synaptic restoration and [^18^F]fallypride-PET/CT indicated functional dopamine release, whilst amphetamine-induced rotation showed significant behavioural recovery. Moreover, histology revealed that the grafted cells matured differently in vivo producing high- and low-tyrosine hydroxylase (TH) expressing cohorts, and only [^18^F]FBCTT uptake was well correlated with differentiation.

**Conclusions:**

This study provides further evidence for the value of in vivo functional imaging for the assessment of cell therapies and highlights the utility of DAT imaging for the determination of early post-transplant cell maturation and differentiation of hESC-mDAs.

## Background

Parkinson’s disease (PD) is a neurodegenerative disease characterised by the progressive loss of dopaminergic neurons projecting from the substantia nigra in the midbrain. This neuronal loss leads to the classical motor symptoms associated with PD including tremors, rigidity and akinesia [1]. Various types of dopamine replacement strategy have been investigated for the treatment of PD including medicinal, surgical and cell replacement strategies. Medicinal strategies are low cost and non-invasive, but long-term treatment is associated with side effects such as dyskinesia and patients eventually develop motor fluctuations [2]. Surgical strategies such as deep brain stimulation have been shown to significantly improve symptoms; however, this requires invasive surgery, with the associated risks of infection or haemorrhage, and has also been associated with long-term side effects including changes in cognition, apathy and anxiety [3]. Cell replacement therapies have been investigated for decades as an alternative treatment for PD and are based on the principle that transplanted dopaminergic neurons can functionally re-innervate the striatum. Early cell replacement studies used cells derived from human foetal ventral mesencephalon, and clinical follow-up studies showed successful re-innervation and functional dopamine release over 10 years after transplantation in some patients [4, 5]. The use of human foetal tissue, however, is not a viable option due to ethical and logistical issues, so alternative, ethical and renewable cell sources are needed to be optimised for transplantation. Results from transplantation studies have shown that transplants with high levels of A9-like dopaminergic neurons are most likely to lead to long-term functional re-innervation [6–10], so the majority of studies have concentrated on midbrain dopaminergic neurons (mDAs) differentiated from human embryonic stem cells (hESCs) [11], induced pluripotent stem cells (iPSCs) and mesenchymal stem cells (MSCs) [12–14]. iPSCs and hESC-derived mDAs have been shown to successfully restore function the denervated striatum in preclinical rat and primate models using standard neuroimaging, behavioural and histological measures [14, 15]. The success of such preclinical studies has led to the use of cell therapies for clinical trials; however, more rigorous preclinical studies are still needed to optimise graft maturation and therapeutic efficacy in vivo. Neuroimaging with PET allows the assessment of different aspects of dopamine neuron function longitudinally in vivo. Examples include [^18^F]FDOPA which measures the biosynthesis of dopamine, [^11^C]raclopride and [^18^F]fallypride which are capable of assessing dopamine release and tropane-derived ligands such as [^18^F]FP-β-CIT, [^18^F]FE-PE2I and [^18^F]FBCTT that have been developed as clinical and preclinical PET radiopharmaceuticals for the quantification of presynaptic dopamine transporter (DAT) expression [16–19].

Neuroimaging is also able to assess the safety of cell transplants by measuring potential tumourigenicity associated with transplanted cells using radiopharmaceuticals such as [^18^F]FDG for metabolism and [^18^F]FLT for proliferation [20]. Such non-invasive imaging measures will be critical for the assessment of mDA cell therapies in clinical trials. In the current study, we have assessed the in vivo maturation, innervation and functional dopamine release of aggregates of immature mDA neurons [21] using neuroimaging and behavioural analysis for 6 months post-transplantation, correlating these measures to graft maturation.

## Materials and methods

### Preparation of hESCs and mDA neuron differentiation

The hESC line HES-3 was obtained from ES Cell International Pte Ltd., Singapore [15]. HES-3 cells were expanded and maintained in mTeSR1™ (STEMCELL Technologies, Canada) medium on tissue culture plates coated with Geltrex (Thermo Fisher Scientific, USA). The cells were passaged at 40 to 50% confluency using ReLeSR™ (STEMCELL Technologies, Canada), in accordance with the manufacturer’s instructions.

The DA neuron differentiation method was derived from a floor plate (FP)-based protocol and modified from Kirkeby et al. [22]. Cells were dissociated with ACCUTASE™ (STEMCELL Technologies, Canada) and cultured on laminin-111-coated (10 μg/ml; BioLamina, Sweden) tissue culture plate in a DIM medium consisting of DMEM/F12:Neurobasal medium (1:1), N2 supplement (1:100), B27 supplement without vitamin A (1:50), penicillin/streptomycin (1:100), GlutaMAX (1:100), non-essential amino acids (1:100) and 100 μM 2-mercaptoethanol (Life Technologies, USA), in the presence of 10 μM Y27632 (Rock inhibitor; Selleckchem, USA), 10 μM SB431542 (SB; Selleckchem, USA), 0.1 μM LDN193189 (LDN; Selleckchem, USA), 200 ng/ml ShhC24II (Shh) (R&D Systems, USA) and 0.8 μM ChIR99021 (ChIR; Selleckchem, USA) for the first 2 days of differentiation. On day 2, the cells were maintained in DIM medium supplemented with SB, LDN, Shh, ChIR and 2 μM Purmorphamine (Pur; Selleckchem, USA) with a medium change every 2 days. On day 8, the cells were differentiated in DIM medium supplemented with Shh, ChIR and 100 ng/ml FGF8 (R&D Systems, USA) with daily medium change. On day 12 of differentiation, the cells were dissociated with ACCUTASE™ and cultured as neurospheres (1 × 104 cells per neurosphere) using AggreWell™800 (STEMCELL Technologies, Canada) in DDM medium consisting of Neurobasal medium, B27 supplement without vitamin A (1:50), penicillin/streptomycin (1:100), GlutaMAX (1:100) and 100 μM 2-mercaptoethanol (Life Technologies, USA), in the presence of Rock inhibitor, FGF8, 10 ng/ml BDNF (STEMCELL Technologies, Canada) and 2 mM ascorbic acid (ASC; Sigma-Aldrich, USA). On day 14, the cells were differentiated in DDM medium supplemented with FGF8, BDNF, ASC, 10 ng/ml GDNF (STEMCELL Technologies, Canada), 1 ng/ml TGFβ3 (STEMCELL Technologies, Canada) and 5 μM DAPT (Selleckchem, USA). On day 16, the neurospheres were embedded in Mebiol® Gel (Cosmo Bio Co. Ltd., Japan) and cultured in DDM medium supplemented with BDNF, ASC, GDNF, TGFβ3, DAPT and CultureOne™ Supplement (1:100; Thermo Fisher Scientific, USA) with medium change every 2 to 3 days. On day 23 of differentiation, the neurospheres were dissociated with ACCUTASE™ to reform smaller-sized neurospheres (5 × 103 cells per neurosphere) using AggreWell™800. On day 25, approximately 4 × 105 cells or 80 neurospheres were delivered per transplantation. Neurospheres not used for transplantation were embedded in Mebiol® Gel and further differentiated in Brainphys™ Neuronal Medium (STEMCELL Technologies, Canada) containing BDNF, ASC, GDNF, TGFβ3, DAPT, CultureOne™ Supplement and NeuroCult™ SM1 Neuronal Supplement (1:50, STEMCELL Technologies, Canada) with medium changed every 3 to 4 days until day 40 of differentiation [23].

### Generation of neurospheres for transplantation

On day 23 of differentiation, the neurospheres were dissociated with ACCUTASE™ to reform smaller-sized neurospheres (5000 cells per neurosphere) for transplantation at day 25. On day 25, approximately 4 × 10^5^ cells or 80 neurospheres were delivered per transplantation.

### Immunofluorescence

Cell aggregates were fixed with 4% paraformaldehyde (Affymetrix, USA) at room temperature (rtp) for 1 h, cryoprotected in 30% sucrose and sectioned by a cryostat before being mounted on glass slides. Sections were permeabilized with 0.5% Triton (Sigma-Aldrich, USA) at rtp for 10 min. They were then blocked with blocking solution consisting of 3% donkey serum (Sigma-Aldrich, USA), 2% CellMaxx™BSA (MP Biomedicals, USA), 0.1% Triton (Sigma-Aldrich, USA) in PBS for at least 1 h before incubating with primary antibodies (1:500 rabbit anti-TH (Pel-Freez, USA) and 1: 1000 mouse anti-Tuj1 (Covance, USA); all diluted in blocking solution) overnight at 4 °C. Cells were incubated with secondary antibodies (1:500 donkey anti-rabbit 488 and 1:500 donkey anti-mouse 555; all diluted in blocking solution) at rtp for 1 h before staining with DAPI solution (all Life Technologies, USA) at rtp for 5 min. Coverslips were mounted with VECTASHIELD Antifade Mounting Medium (Vector Laboratories Inc., USA).

### Western blot and HPLC analysis

At various time points including day 50 of differentiation for HPLC analysis, the neurospheres were dissociated with ACCUTASE™ into single cells. 1 × 106 cells per vial would be snap frozen in liquid N_2_ after washing with PBS. Western blot was carried out using 30 μg of total proteins per sample with primary antibodies (1:1000 rat anti-DAT (Millipore, USA), 1:1000 rabbit anti-TH (Pel-Freez, USA) and 1:10,000 rabbit anti-GAPDH (Cell Signaling, USA)). HPLC analysis was performed as described previously [15].

### 6-Hydroxydopamine lesioning

A total of 75 young female NIH RNu rats were purchased from InVivos Pte Ltd., Singapore, housed 4 to a cage with free access to food and water and held under a 12:12-h light-dark cycle. All surgical operations were performed under ketamine xylazine anaesthesia. Animals received unilateral stereotaxic injections of 6-OHDA.Br (5 μg/μl 4 μl in 0.9% NaCl containing 0.2 mg/ml ascorbic acid) injected into the left medial forebrain bundle (MFB) using a 5-μl Hamilton syringe (− 4.4 mm AP, 1.2 mm ML and 8.6 DV relative to the bregma; 7105KH 5.0 μl, Hamilton Company, Bonaduz, Switzerland, https://www.hamiltoncompany.com/). The injection rate was 0.5 μl/min, and the cannula was left in place for additional 5 min before slowly retracting it.

### Cell transplantation

Cell transplantations were conducted 1 month after 6-OHDA lesion to allow for complete lesion formation. The rats were anaesthetized with a mixture of ketamine and xylazine as described above and then positioned in a stereotactic injection apparatus. Suspensions of concentrated cell aggregates (4 × 10^5^ cells in 4 μl) were loaded into a Hamilton syringe and injected into the left striatum (AP, + 0.8 mm; ML, 2.5 mm; DV, 5 mm). The needle was left in place for 5 min after the injection.

### Radiochemistry

All radiochemical reactions were carried out in anhydrous solvents in closed clear V-vials under air unless stated otherwise. All commercially available reagents were used without further purification unless stated otherwise. [^18^F]fluoride was produced via the ^18^O(*p*,*n*)^18^F reaction using a GE PETrace 800 series cyclotron at the Clinical Imaging Research Center (CIRC) and delivered as [^18^F]fluoride in enriched [^18^O]water. Acetonitrile, ethanol, potassium carbonate and Kryptofix 222 were of analytical grade and bought from Sigma-Aldrich. Water was obtained from an ELGA water purification system (model PF3XXXXM1). Tosyl-Fallypride TFA salt was bought from Bioduro LLC. MBCTT was bought from ABX. V-vials were bought from Fisher Scientific. Accell™ Plus QMA carbonate (46 mg) cartridges, Oasis® HLB light (30 mg) cartridges and ^t^C18 light cartridges were bought from Waters. Gibco™ phosphate-buffered saline (PBS) solution 1× pH 7.4 was bought from Fisher Scientific. Detailed accounts for the radiosynthesis of [^18^F]FBCTT, [^18^F]fallypride and [^18^F]fluoro-l-thymidine can be found in the *Supplementary Materials*.

### Radiosynthesis of [^18^F]FBCTT

[^18^F]FBCTT was prepared by nucleophilic substitution of the MBCTT mesylate precursor with [^18^F]fluoride. [^18^F]FBCTT was formulated for injection in 10% ethanol in phosphate-buffered saline as a colourless solution, pH of 7.4 in a total reaction and purification time of 90 min and with a radiochemical yield of 19–27% (non-decay-corrected, *n* = 10), radiochemical purity > 99% and molar activity of 60–1927 GBq/μmol.

### Radiosynthesis of [^18^F]fallypride

[^18^F]fallypride was prepared by nucleophilic substitution of the tosyl-Fallypride • TFA salt precursor with [^18^F]fluoride. [^18^F]fallypride was formulated for injection in 10% ethanol in phosphate-buffered saline as a colourless solution, pH of 7.4 in a total reaction time of 90 min in a radiochemical yield of 5–19% (non-decay-corrected, *n* = 10), radiochemical purity > 99% and a molar activity of 67–634 GBq/μmol.

### Radiosynthesis of [^18^F]FLT

[^18^F]fluoro-l-thymidine ([^18^F]FLT) was synthesised using the Scintomics GRP™ automated synthesis module (SCINTOMICS GmbH, Fürstenfeldbruck, Germany) equipped with a ready-to-use disposable single-use reagent cassette. [^18^F]FLT synthesis was complete in approximately 60 min providing [^18^F]FLT in 16% non-decay corrected radioactivity yield, in a total volume of 11 ml with a radioactive concentration of 73 MBq/ml at pH 6.0–6.5 with a radiochemical purity of > 99% with a molar activity of 532 GBq/μmol.

### PET imaging

Imaging assessment of lesion success was performed pre-transplantation 4 weeks after 6-OHDA lesion. Animals that displayed greater than 90% lesion were selected for cell transplantation. Imaging was repeated at 1 month, 3 months and 6 months post-transplantation.

NIH RNu rats (*n* = 8 per imaging group) were injected with a solution of either [^18^F]FBCTT (~ 20 MBq in 0.5 ml), [^18^F]fallypride (~ 20 MBq in 0.5 ml) or [^18^F]FLT (~ 5 MBq in 0.5 ml), via the lateral tail vein, and static images were acquired at either 60–90 min, 90–120 min or 30–50 min, respectively, post-injection (static image duration and timings were based on previous dynamic studies to determine the optimal imaging conditions prior to the initiation of the study). The PET and CT images were co-registered to an MRI brain atlas to confirm the anatomical location of the radiopharmaceutical uptake. Uptake of radioactivity was determined by the placement of a region of interest (ROI) over the striatal regions, and the cerebellum uptake was used as a reference region where appropriate. The tissue concentrations are presented as percent injected dose/gram (%ID/g).

### Behavioural analysis

The methamphetamine-induced rotation assay was performed pre-transplantation 4 weeks after 6-OHDA lesion. A dose of 5 mg/kg of methamphetamine was injected intraperitoneally (i.p.), and the rotation behaviour was recorded for 40 min. Animals that displayed greater than 5 ipsilateral rotations/minute were selected for cell transplantation. Behavioural rotation tests were repeated at 1 month, 3 months and 6 months post-transplantation.

### Immunocytochemistry and immunohistochemistry staining

For immunocytochemistry staining, cell aggregates were fixed in 4% PFA (Sigma-Aldrich, Singapore), cryoprotected in 30% sucrose solution, cut on a cryostat and mounted on glass slides. For IHC, the rats were transcardially perfused with 4% PFA; the brains were then removed, post-fixed overnight in 4% PFA and cryoprotected in 30% sucrose. The brain sections were cut with a cryostat and mounted on glass slides. For immunofluorescence, blocking and antibody incubations were performed with a solution of 1% BSA and 0.1% Triton X-100 (Sigma-Aldrich, Singapore) in PBS. The samples were blocked for 1 h at RT, incubated overnight in primary antibody solutions at 4 °C and incubated for 2 h in secondary antibody solutions at RT. After the primary and secondary antibody incubations, the sections were washed four times in PBS. The primary antibodies used are listed in supplemental Table S1. Alexa Fluor 488/568 goat anti-rabbit/mouse IgG (1:400, Invitrogen, Singapore, https://www.thermofisher.com) was used as secondary antibodies for fluorescence labelling. A peroxidase-conjugated goat anti-mouse secondary antibody (Vector Laboratories, Burlingame, CA, USA, https://vectorlabs.com) was used for 3,30-diaminobenzidine (DAB) (Vector Laboratories) labelling, and positive reactions were detected by the avidin-biotin complex (ABC) method (Vector Laboratories). The slices were mounted with fluorescence mounting medium (Dako, Carpinteria, CA, USA, www.dako.com) or DPX (for IHC-DAB staining), and images were obtained with a confocal microscope (LSM710, Olympus, Tokyo, Japan, www.olympus.com).

### Stereological quantification of grafted mDA cells

The total numbers of grafted mDA cells that were immunoreactive for the human nucleus antigen (hNUC), neuronal nuclei antigen (NEUN), TH and FOXA2 were estimated using stereological, unbiased and systematic sampling method described before [24, 25]. Briefly, frozen coronal sections (16 μm thick) were cut and serially collected. Stereological quantification was carried out with every 15th section to cover the entire graft from the rostral focal plane in which the grafted cells started to emerge to the caudal plane where the grafted cells disappeared. The optical images were obtained by an Olympus microscope using a × 40 oil immersion objective. At least eight sections were counted for each graft. The total numbers in the grafts were calculated according to the optical fractionator equation.

### Statistical analysis

Statistical analyses were carried out by using the GraphPad Prism Software (San Diego, CA) and performed using a 2-way ANOVA with post hoc Bonferroni test where appropriate. All data are presented as the mean and standard deviation (mean ± SD) unless otherwise stated.

## Results

### In vitro characterisation of hESC-derived midbrain dopaminergic (mDA) neurons

**Fig. 1:**
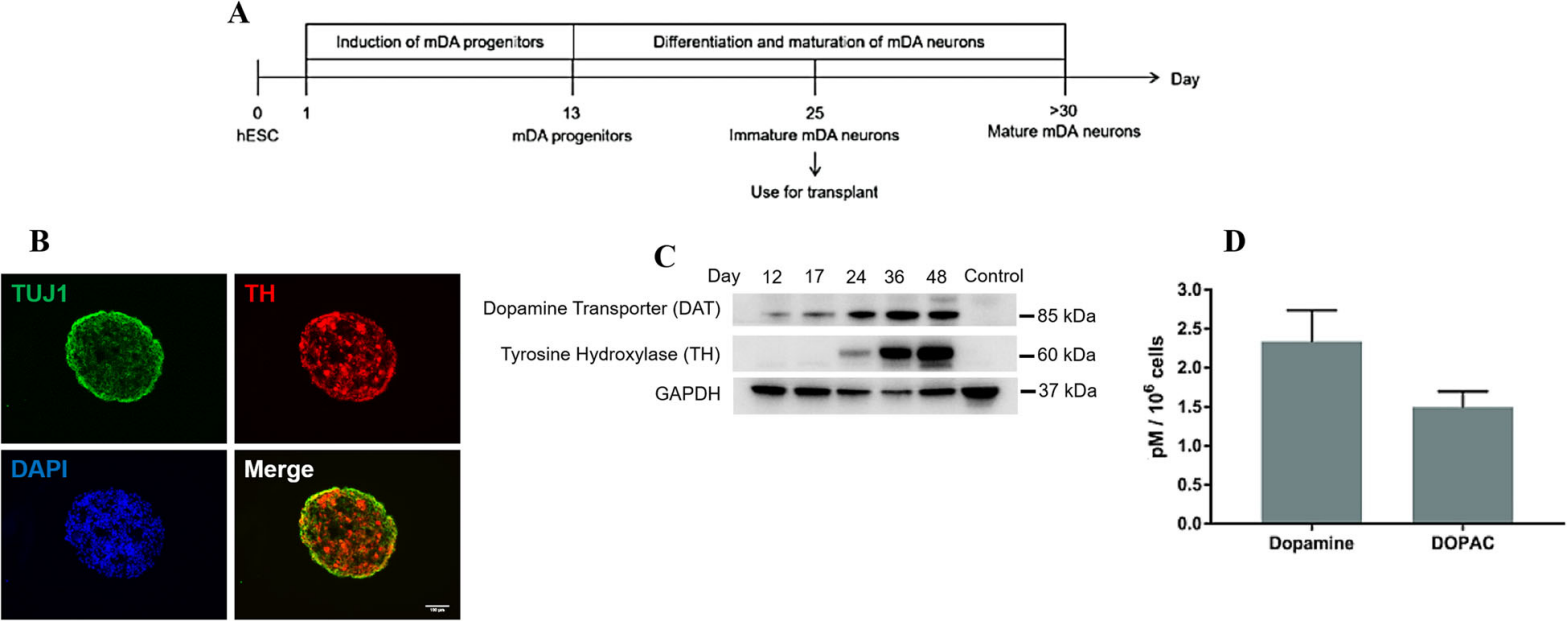
In vitro differentiation of hESCs to generate immature midbrain dopaminergic (mDA) neurons for transplantation. **a** Schematic of mDA neuron differentiation from hESC. **b** Immunofluorescence staining of neurospheres containing immature mDA neurons (TUJ1^+^/TH^+^) at day 48 of differentiation. Scale bar, 100 μm. **c** Western blot showing mDA neuron production of dopamine active transporter (DAT) and tyrosine hydroxylase (TH). **d** HPLC analysis showing immature mDA neurons mature and produce dopamine and DOPAC by day 40 of differentiation

We previously published a systematic study that compared the engraftment efficiency, long-term survival, differentiation, maturation and functional recovery of three groups of human embryonic stem cell (hESC)-derived midbrain dopaminergic (mDA) progenitors and neurons at distinct differentiation stages in a murine model of PD [15]. The three distinct groups of differentiated cells were FOXA2^+^/LMX1A^+^ mDA progenitors, immature TH^+^/NURR1^+^ mDA neurons and more mature mDA neurons with extended neurite outgrowth. We found that transplantation of immature mDA neuronal aggregates was the most efficient in terms of post-transplantation survival ability, neuronal differentiation and maturation in vivo. Therefore, the aggregates of immature mDA neurons derived from hESC were utilised in the current study (Fig. 1a).

The batches of ESC-derived immature mDA neurons were characterised by immunostaining and western blot for standard dopaminergic markers including tyrosine hydroxylase (TH) and dopamine transporter (DAT) as well as HPLC analysis for the production of dopamine and its metabolite 3,4-dihydroxyphenylacetic acid (DOPAC) (Fig. 1b–d). Our results are in agreement with our prior work clearly demonstrating the presence of TUJ1^+^/TH^+^ immature mDA neurons (Fig. 1b). Western blot data showed an increase in the production of TH and DAT over time demonstrating an increase in differentiation efficiency towards the mDA lineage (Fig. 1c), and HPLC analysis confirmed that the hESC-derived immature mDA neurons can successfully mature and produce dopamine (*n* = 3, 2.33 ± 0.4 pM/10^6^ cells) and DOPAC (*n* = 3, 1.49 ± 0.2 pM/10^6^ cells) by day 35 of differentiation (Fig. 1d).

### Quantification of striatal re-innervation by imaging with [^18^F]FBCTT

**Fig. 2:**
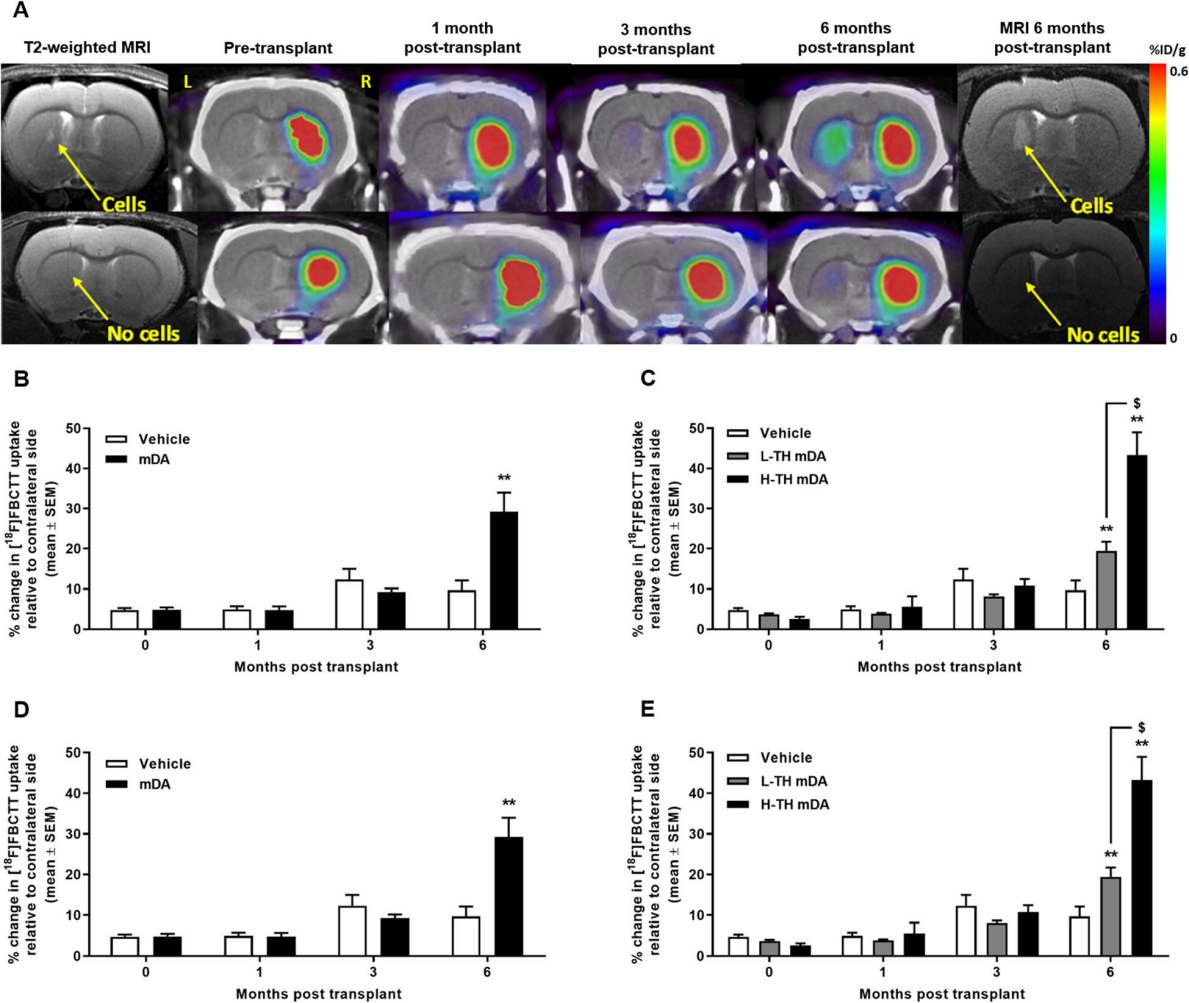
In vivo assessment of striatal re-innervation by imaging with [^18^F]FBCTT. **a** Representative images displaying uptake of [^18^F]FBCTT in the striata of implanted (upper panel) and vehicle-treated (lower panel) rats at each of the time points studied. **b**, **d** Retention of [^18^F]FBCTT measured by longitudinal PET imaging (~ 10 MBq, acquired from 60 to 80 min post-injection). White bars represent data from the vehicle-treated lesioned rat striata, and black bars represent data from the lesioned rat striata implanted with mDAs (*n* = 8, **P < 0.01, 2-way ANOVA with post hoc Bonferroni test, data shown as % change in uptake (**b**) or %ID/g ± SEM (**d**)). **c**, **e** [^18^F]FBCTT uptake in high- and low-TH grafts in vivo. White bars represent data from the vehicle-treated lesioned rat striata, grey bars from the lesioned striata implanted with low-TH mDAs and black bars from the lesioned striata implanted with high-TH mDAs (*n* = 8, 4, 4, **P < 0.01 compared to vehicle, ^$^*P* < 0.05 compared to low-TH, 2-way ANOVA with post hoc Bonferroni test, data shown as % change in uptake (**c**) or %ID/g ± SEM (**e**))

Figure 2a shows the representative images of [^18^F]FBCTT uptake in the striata of implanted and vehicle-treated animals at the time points studied. Figure 2b shows the quantification of [^18^F]FBCTT uptake as a measure of DAT expression in the transplanted and vehicle-treated lesioned rat striata (*n* = 8 rats per group). Imaging assessment of lesion success was performed pre-transplantation, 4 weeks after 6-OHDA lesioning with [^18^F]FBCTT to quantify DAT expression and clearly demonstrated a reduction of greater than 95% (4.3% ± 1.9) compared to the contralateral intact side. One month post-implantation of mDAs, no significant increase in [^18^F]FBCTT was observed in the transplanted striatum (4.9% ± 2.5) compared to the vehicle-treated (5.0% ± 2.3). Small increases in [^18^F]FBCTT retention were observed in both the transplanted striatum (9.3% ± 2.3) and the vehicle-treated striatum (12.3% ± 5.3) at 3 months post-implantation indicating a measure of spontaneous recovery independent of mDA implantation. By 6 months post-implantation, however, a significant and persistent increase in [^18^F]FBCTT retention was observed in the transplanted striatum (29.2% ± 13.4) compared to the vehicle-treated lesioned striatum (9.7% ± 6.9) indicating successful maturation of the implanted mDAs into DAT-expressing mature dopaminergic neurons (**P < 0.01, data shown as mean ± SD % change in [^18^F]FBCTT uptake using the intra-animal contralateral intact striatum uptake as reference).

Figure 2c shows the longitudinal quantification of [^18^F]FBCTT uptake in the low-TH and high-TH transplanted lesioned striata (*n* = 4 per group) compared to vehicle. One month post-successful stereotactic implantation of mDAs, no significant increase in [^18^F]FBCTT was observed in the low-TH transplanted striatum (3.8% ± 0.4) or high-TH transplanted striatum (5.6% ± 4.6) compared to vehicle (5.0% ± 2.3). Small increases in [^18^F]FBCTT retention were observed in the low-TH transplanted striatum (8.1% ± 1.2) and high-TH transplanted striatum (10.7% ± 2.9) but were not significantly different from the vehicle-treated striatum (12.3% ± 5.3) at 3 months post-implantation indicating a measure of spontaneous recovery independent of mDA implantation. By 6 months post-implantation, however, a significant and persistent increase in [^18^F]FBCTT retention was observed in the low-TH transplanted striatum (19.4% ± 4.7, *P < 0.05) and the high-TH transplanted striatum (43.3% ± 9.8, **P < 0.01) compared to the vehicle-treated lesioned striatum (9.7% ± 6.9) indicating successful maturation of the implanted mDAs into DAT-expressing dopamine cells (data shown as mean ± SD % change in [^18^F]FBCTT uptake using the intra-animal contralateral intact striatum uptake as reference).

### Quantification of dopamine release by imaging with [^18^F]fallypride

**Fig. 3:**
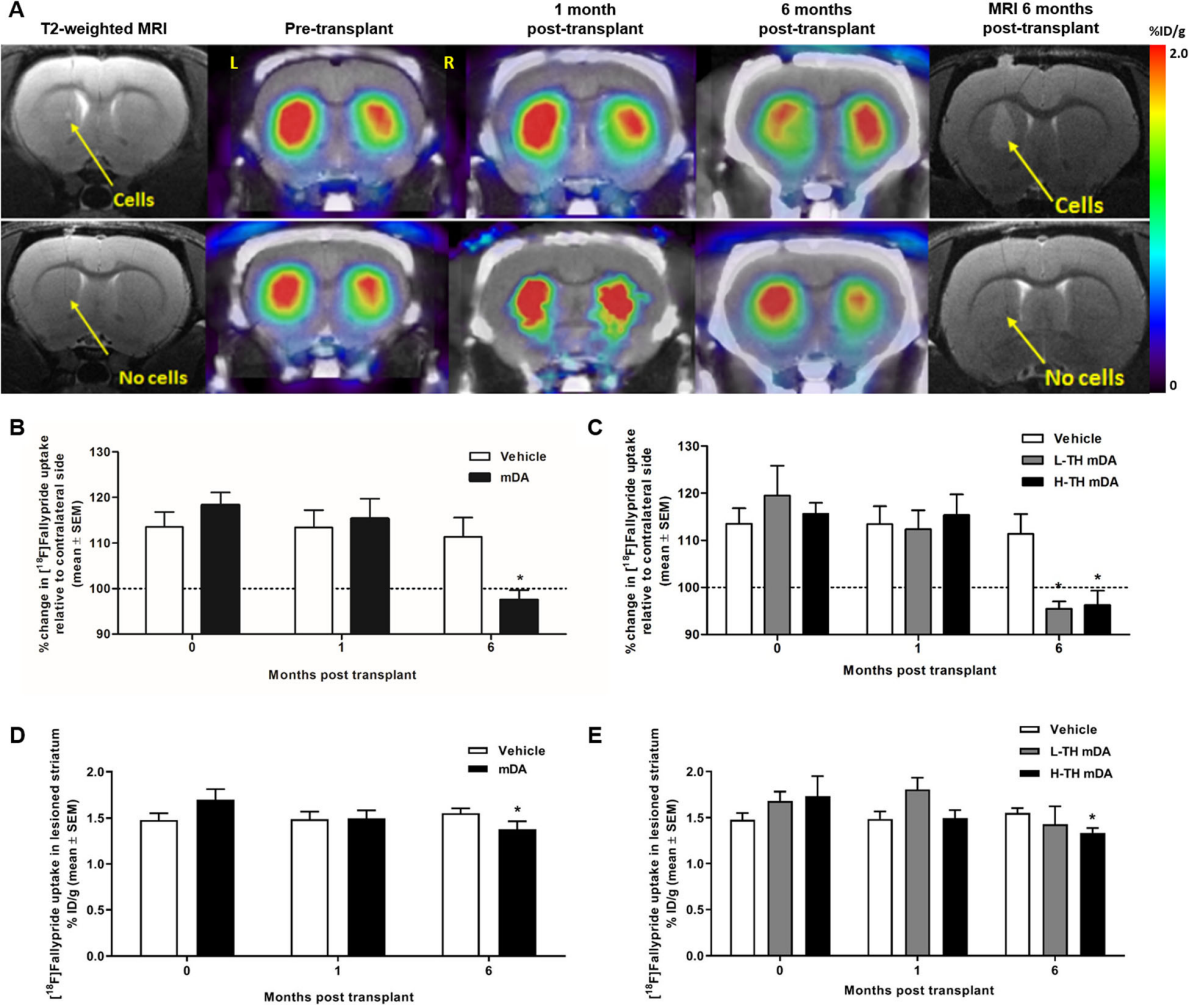
In vivo assessment of dopamine release by imaging with [^18^F]fallypride. **a** Representative images displaying the uptake of [^18^F]fallypride in the striata of implanted (upper panel) and vehicle-treated (lower panel) rats at each of the time points studied**. b**, **d** Retention of [^18^F]fallypride measured by longitudinal PET imaging (~ 10 MBq, acquired from 90 to 120 min post-injection). White bars represent data from the vehicle-treated lesioned rat striata, and black bars represent data from the lesioned rat striata implanted with mDAs (*n* = 8, *P < 0.05, 2-way ANOVA with post hoc Bonferroni test, data shown as % change in uptake (**b**) or %ID/g ± SEM (**d**)). **c**, **e** [^18^F]fallypride occupancy in high- and low-TH grafts in vivo. White bars represent data from the vehicle-treated lesioned rat striata, grey bars from the lesioned striata implanted with low-TH mDAs and black bars from the lesioned striata implanted with high-TH mDAs (*n* = 8, 4, 4, *P < 0.05, 2-way ANOVA with post hoc Bonferroni test, data shown as % change in uptake (**c**) or %ID/g ± SEM (**e**))

Figure 3a shows the representative images of [^18^F]fallypride uptake in the striata of implanted and vehicle-treated animals at the time points studied. Figure 3b shows the longitudinal assessment of [^18^F]fallypride uptake as a measure of post-synaptic D_2_/D_3_ receptor occupancy (*n* = 8 rats per group). Pre-transplant imaging with [^18^F]fallypride showed a significant increase in binding in the lesioned side compared to the contralateral intact side (118.3% ± 8.1) consistent with a profound reduction in dopamine release from the lesioned side. One month post-successful stereotactic implantation of mDAs, no significant change in dopamine receptor occupancy was observed in the transplanted striatum (118.9% ± 9.3) compared to vehicle (113.5% ± 10.7). Six months after implantation, the D_2_/D_3_ receptor occupancy was normalised back to intact levels in the transplanted striatum (97.5% ± 7.2) compared to the vehicle-treated striatum (111.7% ± 11.9) demonstrating functional dopamine release from the transplanted dopamine cells (*P < 0.05, data shown as mean ± SD % change in [^18^F]fallypride uptake using the intra-animal contralateral intact striatum uptake as reference).

Figure 3c shows the longitudinal quantification of [^18^F]fallypride uptake in the low-TH and high-TH transplanted lesioned striata (*n* = 4 per group) compared to vehicle. One month post-successful stereotactic implantation of mDAs, no significant dopamine occupancy was observed in either the low-TH transplanted striatum (123.2% ± 7.9) or the high-TH transplanted striatum (115.4% ± 9.7) compared to vehicle (113.5% ± 10.7). Six months after implantation, the D_2_/D_3_ receptor occupancy was normalised back to intact levels in both the low-TH transplanted striatum (95.5% ± 3.1) and the high-TH transplanted striatum (99.1% ± 8.3) compared to the vehicle-treated striatum (111.7% ± 11.9) demonstrating functional dopamine release from both sets of transplanted dopamine cells (*P < 0.05, data shown as mean ± SD % change in [^18^F]fallypride uptake using the intra-animal contralateral intact striatum uptake as reference).

### Assessment of graft proliferation by imaging with [^18^F]FLT

To quantify the proliferation in the transplanted tissue grafts at the 6-month time point, the transplanted animals were imaged with [^18^F]FLT. No significant difference in [^18^F]FLT uptake was observed between the transplant region and the contralateral intact non-lesioned striatum for either graft.

### Graft survival, neuronal maturation and mDA neuronal differentiation of the transplanted cells at 6 months post-transplantation

**Fig. 4:**
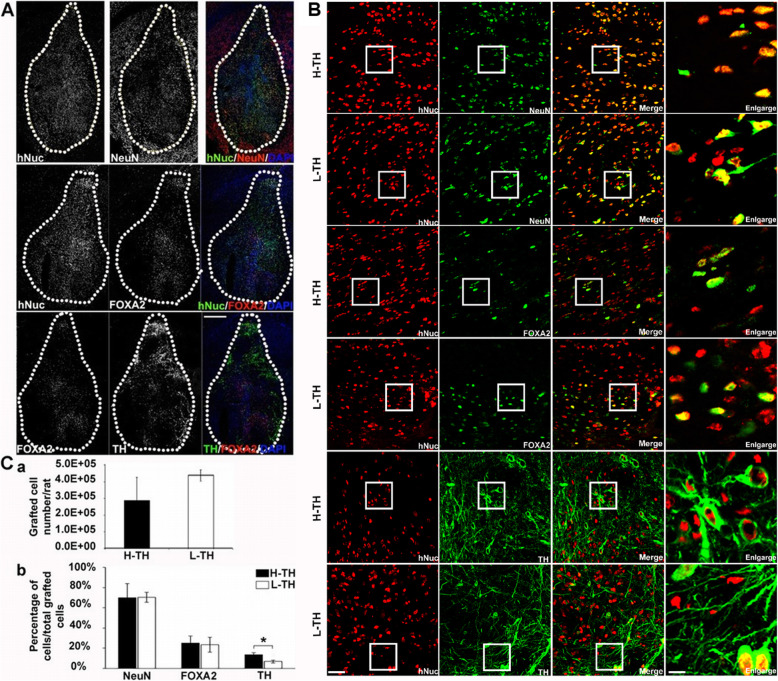
Survival, neuronal maturation and mDA differentiation of the grafted cells. **A** Six months post-transplantation, immunofluorescence staining showed the survival (expression of hNuc) and the neuronal maturation (expression of NeuN) of the transplanted cells in one representative H-TH graft (upper panel). Portion of the grafted cells differentiated into dopaminergic neurons (FoxA2 and TH expression in the middle and lower panels). Dotted lines outline the graft. Scale bar, 500 μm. **B** Enlarged images showed portions of the grafted cells are mature neurons (NeuN expression), midbrain DA progenitors (FoxA2 expression) and DA neurons (TH expression) in high-TH and low-TH grafts respectively. Scale bar, 50 μm. **C** (a) quantification of the surviving transplanted cells in high-TH and low-TH grafts. (b) percentages of NeuN, FoxA2 and TH expressing cells within the high-TH and low-TH grafts (*n* = 3–5, *P < 0.05, 2-way ANOVA with post hoc Bonferroni test, data shown as mean ± SD)

Immunohistochemistry (IHC) was performed to evaluate the survival, neuronal maturation and mDA differentiation of the transplanted cells. The transplanted cells showed substantial survival, as indicated by hNuc staining (Fig. 4A). No sign of overgrowth or necrosis was observed. The majority of surviving grafted cells matured into neurons (as shown by NeuN expression) whilst a portion of these grafted cells differentiated into mDA progenitors (as indicated by FoxA2 expression) and DA neurons (TH expression, Fig. 4A).

We found the percentages of TH^+^ cells in the surviving grafts varied amongst animals. We grouped the grafts into either high-TH grafts (13.33 ± 2.06%) or low-TH grafts (6.65 ± 1.29%; *P < 0.05) (Fig. 4B, C).

Stereological counting of hNuc^+^ cells showed no significant difference in cell survival between the high-TH and low-TH grafts (Fig. 4C (a)) nor any difference in the percentage of NeuN-positive cells between the high-TH grafts (69.88 ± 14.19%) and the low-TH grafts (70.33 ± 4.92%), indicating that the pan-neuronal differentiation was the same (Fig. 4B (a–f); Fig. 4C (b)). Furthermore, no significant difference in the percentage of mDA progenitors (FOXA2-positive cells) was found between high-TH graft (25.08 ± 6.87%) and low-TH graft (23.46 ± 7.39%) (Fig. 4B (g–l); Fig. 4C (b)). These data indicated that the transplanted cells differentiated into neurons, midbrain progenitors and DA neurons with decreased proportions.

### Grafted DA neurons are of midbrain A9 DA neuronal fate

**Fig. 5:**
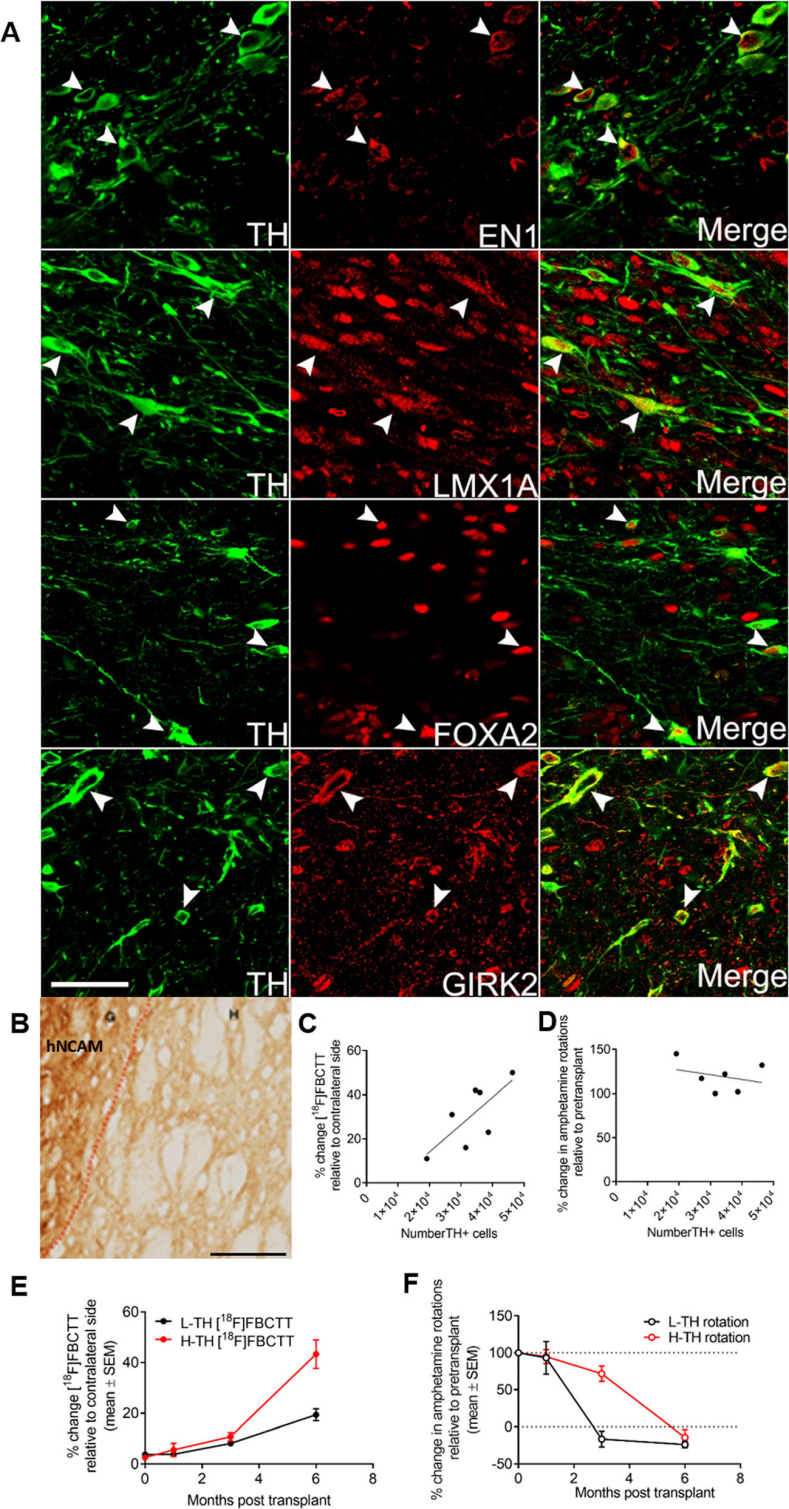
Authentic mDA identity and functional efficacy of the grafted TH^+^ cells and correlations between [^18^F]FBCTT imaging, TH count and behavioural recovery in mDA cell-transplanted PD rats. **a** Immunofluorescence staining for TH (green) and coexpression (red) with EN1 (b), LMX1A (e), FOXA2 (h) and GIRK2 (k). Scale bar, 50 μm. **b** IHC-DAB staining using hNCAM antibody revealed the innervation of the transplanted cells from the graft (G) into the surrounding host striatum (H). Scale bar, 100 μm. **c** A positive linear correlation between [^18^F]FBCTT binding (expressed as % of the contralateral uptake) and the number of TH^+^ cells per graft at 6 months post-transplantation. **d** No correlation between the amphetamine-induced rotation (expressed as % of pretransplant) and the number of TH^+^ cells per graft at 6 months post-transplantation. **e** The change in [^18^F]FBCTT binding (expressed as % of the contralateral uptake) in high-TH versus low-TH grafts at 1, 3 and 6 months post-transplantation. **f** The change in the number of rotations after amphetamine injection longitudinally in high-TH versus low-TH grafts at 1, 3 and 6 months post-transplantation

More markers were evaluated to confirm the midbrain dopaminergic neuron identity of the TH^+^ cells within the grafts. In both high-TH and low-TH grafts, all TH^+^ cells are positive for midbrain neuronal markers EN1, Lmx1a and FOXA2 (Fig. 5a). Furthermore, most of the mature TH^+^ neurons were also positive for A9 neuronal marker, Girk2 (Fig. 5a). Dense hNCAM staining signal revealed the innervation of the transplanted cells into the surrounding striatum of the host brain (Fig. 5b). In contrast, there is little innervation into the host cortex. These results indicate the authentic A9-like mDA identity of the differentiated TH^+^ cells.

### Correlation between quantitative imaging, TH^+^ count and functional efficacy of the high-TH and low-TH grafts

Imaging, TH^+^ cell yield and behavioural tests are currently the main indices used to predict the therapeutic effect of transplanted cells in PD. Thus, we analysed the correlation between these three indices after transplantation. Rotational behaviour induced by amphetamine administration largely corroborated the [^18^F]FBCTT uptake data. One month post-transplantation, neither [^18^F]FBCTT uptake nor behaviour was able to differentiate the low-TH cell-transplanted cohort (3.8 ± 0.4% [^18^F]FBCTT uptake and 95.0 ± 18.7% of rotations relative to pre-transplant) and high-TH cell-transplanted cohort (5.6 ± 4.6% [^18^F]FBCTT uptake and 93.2 ± 38.0% of rotations relative to pre-transplant). By 3 months, however, low-TH grafted animals showed a small 8.1 ± 1.2% increase in [^18^F]FBCTT binding but no behavioural recovery (71.7 ± 20.7% of rotations relative to pre-transplant), whereas high-TH grafted animals showed significant behavioural improvement (− 16.3 ± 18.6% of rotations relative to pre-transplant; Fig. 5c) with only modest (10.7 ± 2.9%) increases in [^18^F]FBCTT binding. At 6 months post-transplantation, both low-TH and high-TH grafted animals showed complete behavioural recovery (− 14.2 ± 21.2% and − 23.9 ± 7.5% of rotations relative to pre-transplant respectively). Nevertheless, the increases in [^18^F]FBCTT binding showed significantly the different uptake between low-TH grafts (19.4 ± 4.7%) and high-TH grafts (43.3 ± 9.8%) (*P < 0.05). These data indicate that both high-TH and low-TH transplanted cells are able to reverse the behavioural deficit but with different efficacies. Overall, no correlation was observed between behaviour and TH^+^ cell number whereas at six months post-transplantation, a positive linear correlation between the percentage of TH^+^ cells and [^18^F]FBCTT binding (*R*^2^ = 0.69) (Fig. 5d) was evident. Taken together, these results indicate that [^18^F]FBCTT imaging is able to quantify the maturation to TH^+^ cells whilst behavioural tests can be saturated by small changes in TH^+^ cell number.

## Discussion

In vivo maturation of cell transplants into TH^+^ dopaminergic cells is a key indicator for PD cell therapy efficacy and much work has been done to improve the chances of cell maturation prior to implantation. Once clinical trials begin, careful monitoring of cell transplant maturation, efficacy and safety after grafting will be paramount. In the current study, we have used neuroimaging and rotational behaviour to longitudinally assess the maturation and efficacy of grafts of immature hESC-mDA aggregates in vivo after transplantation in a rat model of PD. Rotational behaviour induced by amphetamine is often used to measure the therapeutic efficacy of transplanted cells in preclinical models of PD. However, despite amphetamine-induced rotation being widely accepted as an indicator of the extent of motor function impairment based on TH neuronal loss in substantia nigra, how the reduction of amphetamine-induced rotation relates to pre-synaptic recovery is still poorly understood. Previous studies have shown that low-dose 6-OHDA does not affect amphetamine-induced rotation [26]. A negative hyperbolic, but not linear, correlation was found between amphetamine-induced rotation and loss of DA pre-synaptic function with an overt behavioural abnormality emerging when DAT levels decline below 20% of normal. Similarly, our data indicates that a small increase in DAT can lead to significant recovery of rotational behaviour. This implies a limitation in predictive value for the rodent behavioural test with respect to the quality of the transplanted cells in PD and underscores the value of neuroimaging in the pre-clinical assessment of cell therapy. Neuroimaging provides a wide range of measures that are useful for the assessment of cell transplants including quantification of cell survival, dopaminergic function in vivo and the contribution of non-dopaminergic cells [20, 27]. In the current study, imaging clearly showed that hESC-mDAs can safely and successfully restore presynaptic function and dopamine release in the denervated striatum within 6 months but also reliably assess graft maturation/differentiation in vivo. Histology revealed that whilst all grafts survived equally, they matured to produce neurons with different levels of TH in vivo. Many factors affect graft survival and maturation in vivo including host immune response, biochemistry and site of implantation and imaging can provide useful information to help mitigate these risks.

Clinically, both SPECT imaging with [^123^I]FPCIT (DaTscan™) and PET imaging with [^18^F]FP-β-CIT have received the greatest attention in recent years for PD monitoring and assessment of disease progression and initially the wealth of clinical experience offered an ideal scenario for direct translational comparison with the preclinical imaging presented in this work [19, 28, 29]. However, for the current study, the radiosynthesis of the [^18^F]FP-β-CIT yielded particularly poor results (both radiochemical purity and radioactivity yields), and whilst there have been detailed accounts on the optimization of the manufacturing process for [^18^F]FP-β-CIT [30], our manufacture was further hampered by limited access to viable starting materials. Instead, [^18^F]FBCTT was chosen as the DAT PET radiotracer for the current study. [^18^F]FBCTT is a closely related tropane-derivative and could be routinely prepared in good radiochemistry yields (19–27% non-decay corrected yield). Additionally, [^18^F]FBCTT has previously been demonstrated to have a high affinity for DAT and high selectivity over both norepinephrine and serotonin transporters, DAT>NET>>SERT [31, 32].

In the current study, DAT imaging with [^18^F]FBCTT robustly correlated to TH differentiation whereas imaging with [^18^F]fallypride and rotational behaviour did not. [^18^F]fallypride, like raclopride, has been used to measure functional dopamine release after transplant and showed that the grafts in the current study functionally released dopamine 6 months after transplant but was unable to differentiate between the high-TH and low-TH grafts. [^18^F]fallypride has been shown to be a less sensitive measure of striatal dopamine release than [^11^C]raclopride but was used in the current study for logistical reasons where microPET/CT scanning was performed at a site remote from the manufacturing facility and because it allows measurement of D_2_/D_3_ ligand binding in the striatal and extra-striatal regions. However, [^11^C]raclopride has been shown to provide a more sensitive measure of striatal dopamine release after amphetamine challenge [33, 34], and this may have been more successful in differentiating the high-TH and low-TH grafts. Historically, the majority of clinical studies on foetal tissue grafts have used [^11^C]raclopride and [^18^F]FDOPA imaging to assess graft efficacy [35, 36]. [^18^F]FDOPA provides a measure of dopamine biosynthesis and was used for long-term assessment as post-mortem studies have shown that some grafted dopamine neurons express progressively lower levels of DAT and TH decades after transplant, suggesting that the disease process can directly impact the transplanted tissue [17]. However, DAT imaging may be more useful than [^18^F]DOPA in the early stages after transplantation. Imaging of DAT provides a reliable early measure of mDA cell maturation in vivo as DAT is strongly expressed on mature A9-type mDAs [37–44]. Furthermore, during early mDA maturation, interpretation of [^18^F]DOPA images may be complicated by areas of inflammation and/ or on-going dopamine replacement therapies [45–48], whereas DAT imaging is unaffected by these issues.

## Conclusions

Early assessment of cell fate will be important in the clinical assessment of stem cell-derived therapies. This study provides further evidence for the value of in vivo functional imaging for the development of cell therapies and highlights the utility of DAT imaging for the determination of early post-transplant cell maturation and differentiation providing an in vivo measure for cell transplant optimization.

## Data Availability

The datasets used and/or analysed during the current study are available from the corresponding author on reasonable request.

## Abbreviations

% ID/g: Injected dose/gram
6-OHDA: 6-Hydroxydopamine
ABC: Avidin biotin complex
ASC: Ascorbic acid
BDNF: Brain-derived neurotrophic factor
BSA: Bovine serum albumin
ChIR: ChIR99021
CIRC: Clinical Imaging Research Centre
DA: Dopaminergic
DAB: 3,30-Diaminobenzidine
DAT: Dopamine transporter
DOPA: 3,4-Dihydroxyphenylalanine
DPX: Dibutylphthalate Polystyrene Xylene
FBCTT: (1R,2S,3S,5S)-8-Azabicyclo[3.2.1]octane-2-carboxylic acid, 8-(4-fluoro-2-butyn-1-yl)-3-(4-methylphenyl)-methyl ester
FDG: 2-Deoxy-2-[^18^F]fluoro-d-glucose
FDOPA: 6-Fluoro-l-DOPA
FEP2I: (E)-*N*-(3-iodoprop-2-enyl)-2β-carbofluoroethoxy-3β-(4′-methyl-phenyl) nortropane
FGF8: Fibroblast growth factor 8
FLT: [^18^F]fluoro-l-thymidine
FOXA2: Forkhead box protein A2
FP-β-CIT: *N*-3-fluoropropyl-2β-carbomethoxy-3β-(4-iodophenyl) nortropane
GAPDH: Glyceraldehyde 3-phosphate dehydrogenase
GDNF: Glial-derived neurotrophic factor
GIRK2: G protein-activated inward rectifier potassium channel 2
hESCs: Human embryonic stem cells
hESC-DAs: Human embryonic stem cell-derived midbrain dopaminergic neurons
hNUC: Human nucleus antigen
H-TH: High-tyrosine hydroxylase
IC: Immunocytochemistry
IHC: Immunohistochemistry
LDN: LDN193189
L-TH: Low-tyrosine hydroxylase
MBCTT: (1R,2S,3S,5S)-8-Azabicyclo[3.2.1]octane-2-carboxylic acid, 3-(4-methylphenyl)-8-[4-[(methylsulfonyl)oxy]-2-butyn-1-yl]-methyl ester
mDAs: Midbrain dopaminergic neurons
NEUN: Neuronal nuclei antigen
NET: Norepinephrine transporter
PBS: Phosphate-buffered saline
PD: Parkinsons’s disease
PET: Positron emission tomography
PFA: Paraformaldehyde
PUR: Purmorphamine
ROI: Region of interest
RTP: Room temperature and pressure
SB: SB431542
Shh: ShhC24II
SERT: Serotonin transporter
SPECT: Single-photon computed tomography
TGFβ: Transforming growth factor beta
TH: Tyrosine hydroxylase
